# Molecular detection of Epstein-Barr virus among Sudanese patients diagnosed with Hashimoto’s thyroiditis

**DOI:** 10.1186/s13104-023-06399-8

**Published:** 2023-10-19

**Authors:** Marowa N. Hamad, Fuodat I. Mohamed, Mayada M. Osman, Ahlam A. Jadid, Ibtihal K. Abdalrhman, Alaa M. Yousif, Tyseer Alabid, Ali Mahmoud Mohammed Edris, Nouh S. Mohamed, Emmanuel Edwar Siddig, Ayman Ahmed

**Affiliations:** 1 Department of Hematology and immunology, Faculty of Medical Laboratory Sciences, University of Khartoum, Khartoum, Sudan; 2 Molecular Biology Unit, Sirius Training and Research Centre, Khartoum, Sudan; 3 Department of Histopathology and Cytology, Faculty of Medical Laboratory Sciences, University of Khartoum, Khartoum, Sudan; 4 Department of Histopathology and Cytology, Faculty of Applied Medical Sciences, University of Bisha, Bisha, Kingdom of Saudi Arabia; 5 Faculty of Medical Laboratory Sciences, University of Khartoum, Khartoum, 11111 Sudan; 6 Institute of Endemic Diseases, University of Khartoum, Khartoum, 11111 Sudan; 7 Swiss Tropical and Public Health Institute (Swiss TPH), 4123 Allschwil, Switzerland; 8 University of Basel, Petersplatz 1, CH 4001 Basel, Switzerland

**Keywords:** Epstein-Barr virus, Molecular detection, Hashimoto Thyroiditis, Sudan

## Abstract

**Objectives:**

Hashimoto’s thyroiditis (HT) is the most common cause of hypothyroidism. The exact mechanism initiating the development of HT is not yet clear. This study aimed to investigate the correlation between HT and the presence of Epstein-Barr virus (EBV) in a Sudanese population.

**Results:**

EBV-LMP1 was detected in 11.1% of HT cases, which is consistent with previous studies. Studies have reported a wide range of frequencies indicating the presence of EBV in HT, and patients with autoimmune thyroiditis have increased titers of anti-EBV antibodies in their sera compared to healthy subjects. Intrathyroidal EBV-infected B cells may be responsible for the increased risk of development of B-cell lymphoma in the thyroid gland in patients with autoimmune thyroiditis. Our study suggests that regular follow-up is necessary for patients diagnosed with HT and are positive for EBV, as antiviral therapy is not applicable due to the risk of thyroid dysfunction. The study suggests an association between EBV and HT, but causation cannot be determined. The study also highlights the need for further research to determine the viral role and correlate it with the severity and progression of HT.

## Introduction

Autoimmune thyroid diseases (AITDs), such as Hashimoto’s thyroiditis (HT), afflict up to 10% of the world population [1]. While the precise etiology of AITDs remains unclear, it is generally believed that both genetic and environmental factors contribute to their development [2]. Among the environmental factors, viral infections are frequently implicated as playing a role in the development of AITDs [3].

HT, also known as chronic lymphocytic thyroiditis, was named after the Japanese surgeon Hakaru Hashimoto, who first described it in 1912 [4]. This disease affects approximately 5% of individuals at some point in their lives, with the age of presentation typically ranging between 30 and 50 years old, and with females being more affected than males [5, 6]. Histologically, HT is characterized by lymphocytic infiltration in the thyroid gland, with these lymphocytes producing antibodies that trigger an immune response. The most common antibodies found in this disease are anti-thyroperoxidase and/or anti-thyroglobulin antibodies [7]. The etiology of HT remains unclear, although it is believed to be influenced by both genetic and environmental factors. Viral infections are considered to be a major environmental factor that contributes to the development of numerous autoimmune diseases [8].

Epstein-Barr virus (EBV), also known as human herpesvirus 4 (HHV-4), is one of the most common viruses in the Herpes family that affects humans [9]. EBV infects more than 95% of the populations in developed countries, while the percentage increases to 100% in developing countries [10]. The virus is typically transmitted through body fluids, particularly saliva, and is commonly known as the “kissing disease” [9]. The virus has two phases in its life cycle: the lytic phase and the latent phase. In most affected individuals, it takes the latent form in B lymphocytes and in epithelial cells of the nasopharynx. While most individuals infected with EBV are asymptomatic, symptomatic patients may exhibit symptoms such as fever, joint pain, sore throat, diarrhea, and hepatosplenomegaly [10]. In the latent phase, the reactivation of the virus can induce the production of thyroid antibodies and has been implicated in many debilitating autoimmune symptoms [11]. The aim of the current study was to determine the presence or absence of EBV in HT patients by detecting the LMP-1 gene using the PCR technique.

## Materials and methods

### Study design

This is a descriptive, retrospective hospital-based study that was conducted at the Biomedical Research Laboratory, Faculty of Medical Laboratory Sciences, University of Khartoum, Sudan, between February 2020 and June 2021.

### Study population

A total of 135 Formalin-Fixed, Paraffin-Embedded tissues (FFPE) were included in this study after being diagnosed with Hashimoto’s thyroiditis or Grave’s disease at Total Lab Care Clinic. The histopathological examination revealed typical microscopic changes of Hashimoto’s thyroiditis, which consist of lymphoplasmacytic infiltration and lymphoid follicle formation with well-developed germinal centers.

### Preparation of FFPE for DNA extraction

Archival FFPE tissue samples were cut into 12 μm thickness using a Rotary Microtome (Leica, Germany). Under sterile conditions, the tissue sections were transferred into 1.5 ml Eppendorf tubes to avoid cross-contamination. A dewaxing process was performed by adding 1 ml of xylene into each 1.5 ml Eppendorf tube containing tissue sections and left for 30 min at room temperature, allowing the xylene to dissolve the paraffin wax from the tissue. The samples were then centrifuged at 12,000 rpm for 5 min and the supernatant was discarded. The dewaxing process was repeated three times to ensure the complete removal of wax. After dewaxing, a rehydration step was performed using a series of descending grades of ethanol washes starting from 100% to 30 min, followed by 90% and 70% for 3 min each.

### DNA extraction and assessment of DNA quality

DNA extraction from FFPE tissue samples was performed using the QIAamp DNA FFPE kit (Qiagen, Inc, Hilden, Germany) according to the manufacturer’s instructions. The quality of the extracted DNA was assessed using 1% agarose gel electrophoresis and a nanodrop spectrophotometer (ND1000, Houston, TX, USA) following the manufacturer’s guidelines.

### Molecular detection of EBV

Molecular detection of EBV was performed using polymerase chain reaction (PCR). The primer set used was designated for the detection of EBV-LMP-1 gene. The sequence of the sense primer was 5’ CCG AAG AGG TTG AAA ACA AA 3’ and the sequence of the antisense was 5’ GTG GGG GTC GTC ATC ATC TC 3’ [12]. PCR conditions were set on a PCR thermocycler as follows: an initial denaturation step at 95 °C for 5 min, followed by 35 cycles of denaturation at 95 °C for 30 s, annealing at 58 °C for 30 s, and extension at 72 °C for 30 s. A final extension step was performed at 72 °C for 10 min. Positive EBV DNA and distilled water were used as positive and negative controls in each PCR run. PCR products were visualized under UV-light trans-illuminator after being placed into 2.5% agarose gel.

### Statistical analysis

The data analysis was conducted using the Statistical Package for Social Sciences (SPSS version 20.0). The frequency of age groups, as well as the distribution of positive EBV patients among the different age groups, were calculated. The significance of the association between EBV and HT and Graves’ disease was estimated using the Chi-Square test. A p-value of less than 0.05 was considered statistically significant.

## Results

In this study, we investigated 135 FFPETs from females previously diagnosed with HT and gravis disease, with 129 (95.6%) and 6 (4.4%) cases, respectively. The patients’ ages ranged from 20 to 87 years, with a mean age of 52.76 ± 14.97 years. Among the age groups, 63 (46.7%) patients were in the (40–59) years category, which was the majority, while those over 79 years were the least represented, with only 4 (3.0%) cases. The (40–59) and (60–79) years age groups had 44 (32.6%) and 24 (17.8%) patients, respectively.

In terms of residency, the majority of patients were from central Sudan, with 47 (34.8%) cases, followed by northern Sudan, with 25 (18.5%) cases. Southern Sudan had 17 (12.6%) cases. The majority of patients in the study had only a secondary school education, with 48 (35.6%) cases.

Based on the cases family history 28 (20.7%) of the patients had family history of HT disease (Table 1).

**Table 1:** Distribution of the Gravis disease and Hashimoto Thyroiditis by patients’ demographics

	Diagnosis		Total	P value
	Gravis disease	Hashimoto Thyroiditis		
**Patient age group**				
20 to 39 years	1 (4.2%)	23 (95.8%)	24 (17.8%)	0.257
40 to 59 years	5 (7.9%)	58 (92.1%)	63 (46.7%)	
60 to 79 years	0 (0.0%)	44 (100%)	44 (32.6%)	
More than 79 years	0 (0.0%)	4 (100%)	4 (3.0%)	
**Patient residence**				
Central Sudan	2 (4.3%)	45 (95.7%)	47 (34.8%)	0.819
Northern Sudan	2 (8.0%)	23 (92.0%)	25 (18.5%)	
Western Sudan	1 (4.5%)	21 (95.5%)	22 (16.3%)	
Eastern Sudan	1 (4.2%)	23 (95.8%)	24 (17.8%)	
Southern Sudan	0 (0.0%)	17 (100%)	17 (12.6%)	
**Patient educational level**				
Illiterate	0 (0.0%)	15 (100%)	15 (11.1%)	0.109
Khalwa	1 (6.7%)	14 (93.3%)	15 (11.1%)	
Primary School	0 (0.0%)	27 (100%)	27 (20.0%)	
Secondary School	5 (10.4%)	43 (89.6%)	48 (35.6%)	
University	0 (0.0%)	30 (100%)	30 (22.2%)	
**Family history of Hashimoto thyroiditis**				
Yes	0 (0.0%)	28 (100%)	28 (20.7%)	0.241
No	6 (5.6%)	101 (94.4%)	107 (79.3%)	
**Total**	**6 (4.4%)**	**129 (95.6%)**	**135 (100%)**	

The analysis showed that the results of EBV detection were not significantly associated with patients’ demographics and family history of HT, with p-values greater than 0.05. However, the highest frequency of EBV detection was observed among patients residing in southern Sudan, with a rate of 23.5%, followed by northern Sudan (16.0%), central Sudan (10.6%), western Sudan (4.5%), and eastern Sudan (4.2%). The comparison of EBV detection frequency according to residency did not reveal any statistical significance, with a p-value of 0.248. The frequency of patients with EBV and a family history of HT was lower than that of patients with no family history, with rates of 7.1% and 12.1%, respectively. In terms of age group, patients aged between 60 and 79 years had the highest frequency of EBV infection, with a rate of 15.9% (Table 2).

**Table 2:** Distribution of EBV infection by patients’ demographics

	EBV detection		Total	P value
	Positive	Negative		
**Patient residence**				
Central Sudan	5 (10.6%)	42 (89.4%)	47 (34.8%)	0.248
Northern Sudan	4 (16.0%)	21 (84.0%)	25 (18.5%)	
Western Sudan	1 (4.5%)	21 (95.5%)	22 (16.3%)	
Eastern Sudan	1 (4.2%)	23 (95.8%)	24 (17.8%)	
Southern Sudan	4 (23.5%)	13 (76.5%)	17 (12.6%)	
**Patient education level**				
Illiterate	1 (6.7%)	14 (93.3%)	15 (11.1%)	0.148
Khalwa	4 (26.7%)	11 (73.3%)	15 (11.1%)	
Primary School	2 (7.4%)	25 (92.6%)	27 (20.0%)	
Secondary School	7 (14.6%)	41 (85.4%)	48 (35.6%)	
University	1 (3.3%)	29 (96.7%)	30 (22.2%)	
**Family history of thyroiditis**				
Yes	2 (7.1%)	26 (92.9%)	28 (20.7%)	0.358
No	13 (12.1%)	94 (87.9%)	107 (79.3%)	
**Patient age group**				
20 to 39 years	3 (12.5%)	21 (87.5%)	24 (17.8%)	0.529
40 to 59 years	5 (7.9%)	58 (92.1%)	63 (46.7%)	
60 to 79 years	7 (15.9%)	37 (84.1%)	44 (32.6%)	
More than 79 years	0 (0.0%)	4 (100%)	4 (3.0%)	
**Total**	**15 (11.1%)**	**120 (88.9%)**	**135 (100%)**	

The analysis of EBV detection based on the patients’ previous diagnosis revealed that none of the patients diagnosed with Gravis disease had a positive result for EBV. On the other hand, all patients who tested positive for EBV were diagnosed with HT. However, no statistically significant association was found between EBV detection and HT diagnosis, with a p-value of 0.486 (Table 3).

**Table 3:** Association between EBV infection, HT, and Gravis disease

		Histopathological diagnosis		Total	P value	Odds ratio	95% Confidence Interval	
		HT	Gravis disease				Lower	Upper
EBV detection	Positive	15 (100%)	0 (0.0%)	15 (11.1%)	0.486	1.056	1.010	1.097
	Negative	114 (95.0%)	6 (5.0%)	120 (88.9%)				
	Total	129 (95.6%)	6 (4.4%)	135 (100%)				

## Discussion

Hashimoto thyroiditis (HT) is the most common cause of hypothyroidism [6]. The exact mechanism that initiates the development of HT has not yet been clarified. However, multiple factors are thought to play a critical role in the development of HT, including genetic, viral diseases, environmental factors, and others [13]. Among the viruses associated with the induction of several autoimmune diseases, EBV is considered the most common. Once a patient is infected with the virus, it never goes away, as the virus can be dormant and reside inside B-cells until it finds a suitable trigger for activation [9]. In this study, the correlation between HT and the presence of the EBV genome was examined. Although a growing body of studies has investigated this correlation, findings are greatly contradictory. A wide range of frequencies indicating the presence of EBV in HT has been reported [14, 15]. Furthermore, several studies show that patients with autoimmune thyroiditis have increased titers of anti-EBV antibodies in their sera compared to healthy subjects [16]. Intriguingly, thyrotoxicosis can develop immediately after infectious mononucleosis due to primary EBV infection, and autoimmune hypothyroidism can develop in association with acute EBV infection [17]. Intrathyroidal EBV-infected B cells could be the source of the monoclonally expanded B cells in the thyroid gland in autoimmune thyroiditis and might be responsible for the increased risk of B-cell lymphoma development in the thyroid gland in patients with autoimmune thyroiditis [18, 19].

In the present study, EBV LMP1 was detected in approximately 11.1% of our samples, which is in concordance with Janegova and colleagues, who investigated the role of EBV in the development of autoimmune thyroid disease by examining surgical specimens of Graves’, Hashimoto’s, and multinodular goiter for the presence of EBV-Latent membrane protein using immunohistochemistry. Interestingly, their results showed that virus-encoded small nuclear non-polyadenylated RNAs (EBER) were detected using in situ hybridization technique. EBER nuclear expression was well-detected in 80.7% of Hashimoto’s thyroiditis cases and 62.5% of Graves’ disease cases, with a positive correlation between LMP1 and EBER positivity in all Hashimoto’s thyroiditis LMP1-positive cases [15]. Furthermore, Swanson-Mungerson and colleagues used transgenic mouse models to investigate whether EBV plays a role in the induction of autoimmune thyroid disease. Their study suggests that B cells expressing the EBV-encoded protein latent membrane protein 2 A (LMP2A) bypass normal tolerance checkpoints and enhance the development of autoimmune diseases. Their evidence from transgenic mouse models supports a paradigm in which LMP2A could promote autoimmune development. Interestingly, this novel model provides a framework to test potential mechanisms by which EBV could promote the development of autoimmune responses and might enable the identification of strategies to treat EBV-associated autoimmune diseases [20]. Additionally, as HT has been reported by many clinical cohort studies to have an increased risk of lymphoma by 3–4 folds, a regular follow-up is mandatory for patients diagnosed with HT who are positive for EBV since antiviral therapy cannot eradicate the EBV, and the risk of thyroid dysfunction is very high among patients on antiviral therapy [21].

In conclusion, this study provides evidence for the presence of EBV-LMP-1 gene in a subset of patients with Hashimoto thyroiditis in Sudan. Our findings support the growing body of literature suggesting a potential role for EBV in the development of autoimmune thyroid diseases, including HT. Regular follow-up for patients diagnosed with HT and positive for EBV is important for proper management, as antiviral therapy is not applicable in these patients due to the risk of thyroid dysfunction. Future studies are needed to determine the causal relationship between EBV and HT and to investigate the potential mechanisms underlying this association.

### Limitations

- The study only demonstrated the expression of the EBV-LMP1 gene in patients with HT.
- The study could not determine if EBV infection from the beginning led to the development of HT in the thyroid gland.
- The sample size of the study was relatively small and limited to a specific population in Sudan.
- The generalizability of the findings to other populations may be limited.
- Future studies with larger sample sizes and diverse populations are needed to confirm the results.

## Data Availability

The datasets used and/or analyzed during the current study are available from the corresponding author on reasonable request.
